# Observational Constraints on Cloud Feedbacks: The Role of Active Satellite Sensors

**DOI:** 10.1007/s10712-017-9452-0

**Published:** 2017-11-30

**Authors:** David Winker, Helene Chepfer, Vincent Noel, Xia Cai

**Affiliations:** 1 MS/475, NASA Langley Research Center, Hampton, VA 23681 USA; 2 LMD/IPSL, CNRS, UPMC, University of Paris 06, 75252 Paris, France; 3 Laboratoire d’Aérologie, CNRS, 31400 Toulouse, France; 4 Science Systems and Applications, Inc (SSAI), Hampton, VA 23666 USA

**Keywords:** Cloud feedback, Satellite lidar, Radar, Deep convection, Shallow clouds

## Abstract

Cloud profiling from active lidar and radar in the A-train satellite constellation has significantly advanced our understanding of clouds and their role in the climate system. Nevertheless, the response of clouds to a warming climate remains one of the largest uncertainties in predicting climate change and for the development of adaptions to change. Both observation of long-term changes and observational constraints on the processes responsible for those changes are necessary. We review recent progress in our understanding of the cloud feedback problem. Capabilities and advantages of active sensors for observing clouds are discussed, along with the importance of active sensors for deriving constraints on cloud feedbacks as an essential component of a global climate observing system.

## Introduction

Equilibrium climate sensitivity (ECS)—the sensitivity of global-mean surface temperature to a doubling of CO_2_—has become an organizing concept of climate science which reflects many aspects of climate change including changes of the hydrologic cycle and regional climate. ECS was first estimated in the late 1970s, based on clever inferences from two early global circulation models, with a range of 1.5–4.5 °C (Charney et al. 1979). Since then, our understanding of the climate system has improved markedly and much effort has been applied to reducing this range of uncertainty. However, in the most recent report of the IPCC, the ECS is still judged to be “likely in the range 1.5–4.5 °C” (IPCC 2013), illustrating that the uncertainties in ECS have proven to be remarkably resilient. Much progress has been made, however, in the identification and understanding of the processes that control climate sensitivity (Stevens et al. 2016a). A reduction in the range of ECS—particularly at the high end where the risks are greatest and the economic benefits of better information are largest—would be tremendously valuable in improving our abilities to better plan for climate change (Cooke et al. 2013; Neubersch et al. 2014) and would help determine the levels of greenhouse gas emissions compatible with a global warming target of 1.5 or 2 °C.

Estimates of ECS based on the historical surface temperature record tend to fall at the low end of the IPCC range (Otto et al. 2013; Lewis and Curry 2015; Forster 2016), while observational tests of processes relevant to cloud feedbacks tend to point toward the high end of the range (e.g., Sherwood et al. 2014; Su et al. 2014). This inconsistency has been a focus of research efforts in recent years, and questions have been raised as to whether the historical record is fundamentally unsuitable for constraining ECS (Knutti and Hegerl 2008). In spite of these difficulties, techniques of analyzing model output have become increasingly sophisticated over the last decade and it is now well established that the largest source of model diversity in ECS is due to cloud feedbacks (Bony et al. 2006; Webb et al. 2013). The large spread in model ECS can be traced to the complexity of the processes involving clouds and the difficulty of realistically representing these processes in global models. The occurrence and properties of clouds are the result of multiple competing processes taking place over a wide range of space and time scales. Many processes must be highly parameterized in the global models used for climate studies, and there are questions as to whether the key processes are captured sufficiently well. Much has been learned from high-resolution eddy-scale models, but interactions across spatial and temporal scales are also important and models are only beginning to reach the point where these scale interactions can be directly simulated.

Providing observational constraints on cloud feedbacks poses a significant challenge. Clouds have a large impact on Earth’s radiation budget with global-mean shortwave (SW) and longwave (LW) cloud radiative effects estimated at − 47.1 and + 26.5 W/m^2^, respectively, resulting in a net cooling of about 20 ± 4 W/m^2^ (Loeb et al. 2009). Because the radiative effects of clouds are large relative to the radiative forcing of 3.7 W/m^2^ expected from a doubling of CO_2_ (Myhre et al. 2001), even small forced changes in clouds can act to significantly enhance or mitigate greenhouse gas warming. Observing these small changes requires an observing system which is highly accurate and stable over the decades required for trends to emerge from the noise of climate variability.

The international “A-train” constellation, built around a core consisting of the Aqua, CALIPSO, and CloudSat satellites, represents a major advance underway since the late 1990s in our ability to observe Earth’s climate system. The A-train has provided an unprecedented, comprehensive set of observations of clouds, aerosols, and atmospheric state (L’Ecuyer and Jiang 2010) which have significantly advanced our understanding of climate. But more progress is needed. In this paper, we address the question of how observations can be used to reduce current uncertainties in cloud feedbacks. In the next section, we briefly summarize the current understanding of cloud feedbacks. Section 3 reviews some of the strengths and weaknesses of lidar for monitoring cloud changes. In Sect. 4, we review some of the approaches used to determine cloud feedbacks from satellite sensors, difficulties posed by non-ideal instruments, and discuss new opportunities presented by the emerging record from active satellite sensors. In particular, what could be learned from extending the current record obtained from the active sensors in the A-train constellation to multidecade time scales. Finally, Sect. 5 presents an outline of the satellite observations beyond the A-train necessary to continue advancing our understanding of cloud processes and feedbacks.

## What Do We Know? What Do We Still Need to Know?

Cloud feedbacks, and tropical low-cloud feedbacks in particular, have been identified as the dominant source of uncertainty in model estimates of climate sensitivity (Bony and Dufresne 2005; Vial et al. 2013). Reducing these uncertainties has proven difficult, in part due to the difficulty of quantifying the factors controlling clouds and their response to a warming climate. But rapid progress has been made in developing techniques to interrogate model simulations and in delineating the cloud types and factors responsible for both feedbacks and their uncertainties. Plausible physical mechanisms have been proposed. Ultimately, climate models must be tested against observations and model parameterizations must capture the key physical mechanisms operating in the real world.

The effect of clouds on outgoing radiative fluxes is referred to as the cloud radiative effect (CRE). This can be computed as the difference between all-sky fluxes and fluxes from cloud-free scenes, or between cloudy and cloud-free scenes:

1 $${\text{CRE}} = F_{\text{clear}} - F_{\text{all-sky}} = {\text{CF}}(F_{\text{clear}} - F_{\text{cloud}} )$$

where CF is the total cloud fraction and *F* refers to the net upwelling minus downwelling radiative fluxes at the top of the atmosphere (TOA). A “cloud radiative feedback” is the perturbation of outgoing radiative fluxes due to the changes of clouds in response to climate warming. While the change in CRE over time is equal to the change in clear-sky minus all-sky radiative fluxes, the cloud radiative feedback is the change in outgoing radiation due to clouds alone. Clouds have a masking effect, in that they reduce the contribution of changes in lower-level temperatures, surface albedo, and moistening to the outgoing radiative fluxes from those which would occur if the clouds were not present. Therefore, cloud feedbacks are estimated by adjusting the change in CRE for cloud masking of the temperature and water vapor feedbacks. This is commonly done using the radiative kernel approach pioneered by Soden et al. (2008). In climate models, longwave (LW) cloud feedbacks are driven by changes in mid- and high clouds (Zelinka et al. 2016). The largest feedback uncertainties, however, are associated with the shortwave (SW) feedbacks of shallow marine clouds (Bony et al. 2006; Soden and Held 2006; Vial et al. 2013).

### What Observable Changes Do We Expect Under Climate Change?

Cloud radiative feedbacks result from changes in cloud fraction, height, or optical depth in response to rising greenhouse gas concentrations. Changes in optical depth can arise from changes in cloud liquid water path or by changes in thermodynamic phase. Zelinka et al. (2016) developed a decomposition of cloud feedback in terms of changes in macroscopic cloud properties. Their analysis of the feedbacks predicted by an ensemble of climate models shows low-cloud feedbacks (> 680 hPa) are dominated by a robust positive net cloud amount feedback arising from SW effects, with essentially zero altitude feedback from low clouds. A negative low-cloud optical depth feedback is found, primarily at high latitudes, which may involve changes of cloud phase from ice to water as the atmosphere warms. In contrast, non-low clouds (mid-level and high clouds above 680 hPa) show a robust and positive altitude feedback due to LW CRE. The LW and SW components of the cloud amount and cloud optical depth feedbacks of non-low clouds exhibit relatively large model diversity but tend to compensate each other. The cloud altitude, amount, and optical depth feedbacks vary geographically in complex patterns, compensating each other some places and reinforcing in others.

Determining the sensitivities of these cloud responses to warming surface temperatures, in the presence of large natural variability, represents one of the main challenges in characterizing and constraining cloud feedbacks. The many uncertainties associated with model estimates of cloud feedbacks motivate approaches based on observations. These can be roughly divided into studies analyzing observed trends over the satellite era and those which try to derive constraints on long-term cloud feedbacks from observations of interannual variability.

### How Might We Diagnose These Changes?

Models robustly predict that high clouds will rise in a warming climate such that cloud top temperatures remain nearly constant, producing a positive LW feedback (Hartmann and Larson 2002; Zelinka and Hartmann 2010). The rise in cloud height occurs at all latitudes and in the extratropics is associated with increasing height of the tropopause. This appears to be a consequence of the fundamental mechanism underlying radiative-convective equilibrium: To maintain equilibrium, upward convective ascent must be matched by radiatively driven clear-sky subsidence. Thus, the vertical profile of clear-sky diabatic cooling acts as a control on the vertical development of tropical deep convection. Using observations from multiple A-train sensors, Zelinka and Hartmann (2011) found support for this mechanism by regressing monthly mean anomalies against sea surface temperature (SST). Li et al. (2012), in a similar study, point out that cloud profiles, as can obtained from active sensors, are a more appropriate indicator of the cloud detrainment level than cloud top temperature derived from passive sensors, and would provide better quantification of the SST–cloud top relationships.

Other studies have looked in observations for the trends in tropical cloud heights predicted by models. Norris et al. (2016) analyzed the 30-year (1983–2009) ISCCP and PATMOS-x datasets, both based on observations from a series of operational geostationary and polar orbiting satellites. They found it necessary to remove systematic biases due to satellite orbit drifts and sensor calibration differences, with the result of also removing global-mean variability and any climate signal which is geographically correlated with the biases removed (Evan et al. 2007; Norris and Evan 2015). They found changes in cloud height consistent with rising cloud altitude, but uncertainties in the datasets used precluded a quantitative estimation of the magnitude. Analysis of the two different cloud datasets—derived from similar sets of satellites, although using different algorithms—show significantly different patterns of changing cloud vertical occurrence, illustrating the uncertainties involved in using the existing long-term passive cloud climatologies for trend analysis.

Attention has focused recently on a more fundamental problem in deriving cloud feedbacks from observed trends. It is known that the SST change expected from rising CO_2_ levels is more uniform than that from natural variability. Recent work shows that climate feedbacks are not constant but vary over the historical period (Armour et al. 2013; Xie et al. 2016; Gregory and Andrews 2016; Rugenstein et al. 2016) and that this comes, in part, from the dependence of cloud feedbacks on patterns of surface temperature change. In particular, Zhou et al. (2016) find the spatial patterns of surface warming have varied in a way that cloud feedbacks over recent decades are significantly more negative than long-term feedbacks. Su and Jiang (2013) provide an illustration of this, using observations from CALIPSO and CloudSat to show that El Nino events with different patterns of SST change can produce nearly opposite responses in the vertically resolved tropical cloud occurrence and cloud water content. They further diagnose that a significant part of the cloud response to SST changes is mediated by changes in large-scale circulation. There are also longer-term modes of climate variability operating on decadal and multidecadal time scales such as the Atlantic multidecadal oscillation (Enfield et al. 2001) and the interdecadal Pacific oscillation (Folland et al. 1999). These modes are associated with patterns of SST change distinct from those of both ENSO and global warming, further complicating the task of relating cloud feedbacks estimated from current observations to long-term feedbacks.

Given the difficulty of deriving constraints on long-term cloud feedbacks from observed trends, there has been a recent focus on relating the observed variability of shallow clouds—on time scales ranging from weekly to interannual—to controlling factors such as SST and the strength of the marine boundary layer inversion. In the so-called emergent constraints approach (Klein and Hall 2015), observed relationships between clouds and their controlling factors are used to constrain long-term feedbacks from models under the assumption there is a relationship between simulated present day and centennial scale variabilities. In one recent paper, Zhai et al. (2015) use observations from AIRS and CALIPSO–CloudSat to compute the sensitivity of marine low-cloud cover to changes in SST in regions of large-scale subsidence. They find the sensitivities at interannual and centennial scales are correlated in models. In a comparison of observed and modeled seasonal sensitivities, the models with seasonal sensitivities consistent with observations were found to have high ECS, whereas models inconsistent with the observations tended to have low ECS. Marine low-cloud cover and shallow cloud feedbacks are not uniquely determined by SST however. Some models showed a strong link between low cloud cover and SST, but in other models the link was much weaker, indicating the importance of other controlling factors such as tropical inversion strength, lower tropospheric stability, water vapor, and variations in atmospheric circulations. Thus, there is still a need to confirm these results with long-term observations.

Qu et al. (2015) took a different approach to deriving long-term constraints from observed interannual variability. They used observations to estimate the sensitivity of shallow clouds to their controlling factors and then applied these sensitivities to the changes in the large-scale environmental simulated in future climates. The advantage of this approach is it does not rely on climate model simulations of clouds, but instead uses the more trustworthy predictions of how the large-scale controlling factors will change.

A number of other recent studies use one or both of these short-term variability approaches (Brient and Schneider 2016; Myers and Norris 2016; McCoy et al. 2017). Uncertainties in feedbacks estimated from these studies are still relatively large, but a meta-analysis by Klein et al. (2017, this issue) is able to derive a useful constraint on global models which indicates negative and near-zero tropical cloud feedbacks are unlikely. Even if these approaches are able to provide constraints on model feedbacks, however, they don’t provide a straightforward path to improving the representation of cloud processes in models. Observational studies are still required to identify and constrain the mechanisms responsible for cloud feedbacks.

## Observing Clouds with Active Sensors

While research over the last decade has greatly increased our understanding of cloud feedbacks, uncertainties in their magnitude have not been significantly reduced. Given the variety of approaches which have been explored, what are the prospects for reducing the current uncertainties and how can the (relatively new) availability of observations from active sensors contribute?

Satellite observations over multiple decades are necessary to directly detect the emergence of forced changes and separate them from natural variability. A National Institute of Standards and Technology (NIST) report (Ohring 2004) has identified stability and accuracy requirements on measurements used to characterize climate change trends. Requirements on cloud measurements were tied to current abilities to monitor solar irradiance and outgoing TOA LW fluxes to within a few tenths of a W/m^2^ per decade. Climate monitoring requirements identified for clouds, necessary to constrain cloud feedbacks, are summarized in Table 1. Requirements on the accuracy of global-mean cloud cover and global-mean cloud top height were set at 1% and 150 m, respectively, with requirements on measurement stability of 0.3%/decade and 30 m/decade. These requirements call for observations with exceptional stability, and the calibration uncertainties of the satellite radiometers in current use significantly increase the time to detect cloud trends relative to a perfect instrument (Wielicki et al. 2013; Shea et al. 2017).

**Table 1:** NIST requirements for climate-accuracy cloud observations

Parameter	Accuracy	Stability
Cloud top temperature	1 K	0.2 K/decade
Cloud top cover	1%	0.3%/decade
Cloud top height	150 m	30 m/decade
Cloud base height	500 m	100 m/decade

CALIPSO (Winker et al. 2010) and CloudSat (Stephens et al. 2002), launched together in 2006, have provided our first experience with active profiling of clouds from space. These instruments have now acquired more than 11 years of global cloud profiles over a period which includes three ENSO cycles—long enough to sufficiently characterize the mean state of clouds in the early twenty-first century. Lidar offers the necessary accuracy, sensitivity, and long-term stability to monitor the small trends in cloud cover and altitude expected under climate change and provide constraints on cloud radiative feedbacks.

Passive cloud retrievals are based on relatively simple forward models—involving assumptions on atmospheric vertical structure—and large errors and ambiguities can ensue when these assumptions are violated in the real atmosphere (Stephens and Kummerow 2007). Whereas passive retrievals are limited to retrieval of a single effective cloud height, lidar provides a profile of the vertical distribution of cloud—including multilayer clouds—down to the altitude where the return signal is extinguished by cloud attenuation. Lidar cloud altitude is directly measured from the laser pulse time-of-flight (Winker et al. 2007). Measurement of the time delay is inherently accurate, and independent of radiometric calibration. Knowledge of the distance from the satellite to the ocean surface provides an additional constraint. The satellite orbit altitude is determined to within 30 m using the global positioning system (GPS), so that the range from satellite to the ocean surface is known independently of laser timing. Any potential timing drifts can be identified by an apparent change in the time delay from emission of the laser pulse to detection of the laser return from the ocean surface. GPS orbit determination allows one to constrain errors in lidar altitudes due to a potential instrument clock drift to about 1 m absolute long-term accuracy.

**Fig. 1:**
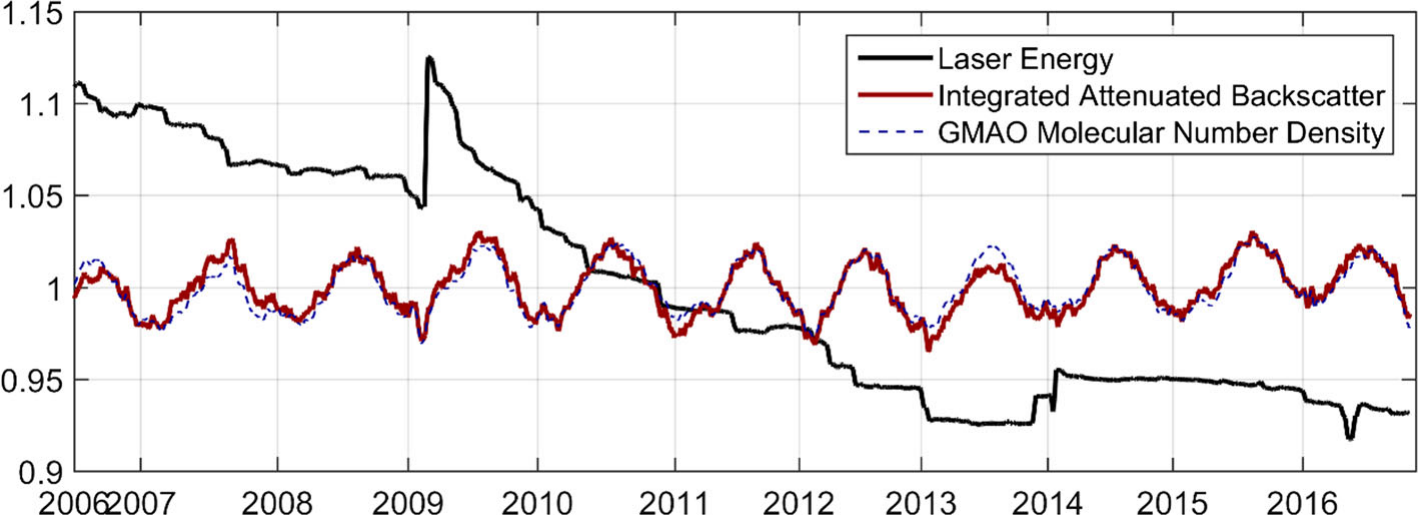
Normalized trends of laser total pulse energy, mid-stratospheric 532 nm attenuated backscatter signals integrated between 25 and 40 km altitude, and molecular number density averaged over the same altitude range

Clouds are detected in the lidar backscatter profile via the contrast between returns from clouds and the molecular atmosphere. As this is a relative measurement, cloud detection is largely independent of potential calibration errors. For those measurements which do depend on radiometric calibration, lidar returns from the mid-stratosphere are referenced to molecular density profiles computed from global re-analysis products—essentially using the mid-stratosphere as a calibration target (Powell et al. 2009). Radiometric calibration of Cloud-Aerosol Lidar with Orthogonal Polarization (CALIOP) is currently performed using lidar returns between 35 and 40 km altitude, where aerosol contributions are negligible. Experience with CALIPSO has shown these calibrations have excellent long-term stability, as illustrated in Fig. 1. The red curve shows a time series of CALIOP 532 nm attenuated backscatter (calibrated profile data) integrated from 24 to 40 km, where the lidar backscatter is dominated by molecular scattering, and averaged over 50°S–50°N. The blue dashed curve is the normalized molecular number density from the NASA Global Modeling and Assimilation Office Forward Processing for Instrument Teams (GMAO-FP-IT) analysis product over the same altitude range, interpolated to the CALIPSO ground track. The black curve shows the time history of 532-nm laser pulse energy over the mission. Pulse energy has varied over time due to the loss of pump diodes, adjustments to the laser, and a switch between primary and backup lasers in March 2009. The calibration scheme is able to accurately correct the profile data for these variations in pulse energy, producing a stable long-term record.

With high sensitivity and high-resolution vertical profiling capability, lidar provides the most rigorous observations of cloud fraction and cloud top height. Cloud profiling radar provides complementary information on the vertical distribution of optically thick clouds. CALIOP has been used extensively to identify and characterize biases in passive cloud height retrievals due to broken or multilayer clouds, or when clouds lie underneath temperature inversions (Holz et al. 2008; di Michele et al. 2013). In particular, CALIOP and CloudSat together have been used to illustrate the biases in the cloud top pressure-optical depth (CP-*τ*) diagrams derived from passive sensors due to ambiguities in passive cloud height retrievals (Marchand et al. 2010; Mace and Wrenn 2013). Relative to using passive sensors alone, Stephens et al. (2017) illustrate the improved ability to partition CRE by cloud type using active and passive sensors together, primarily due to the ability of active sensors to identify and characterize multilayer cloud situations.

**Fig. 2:**
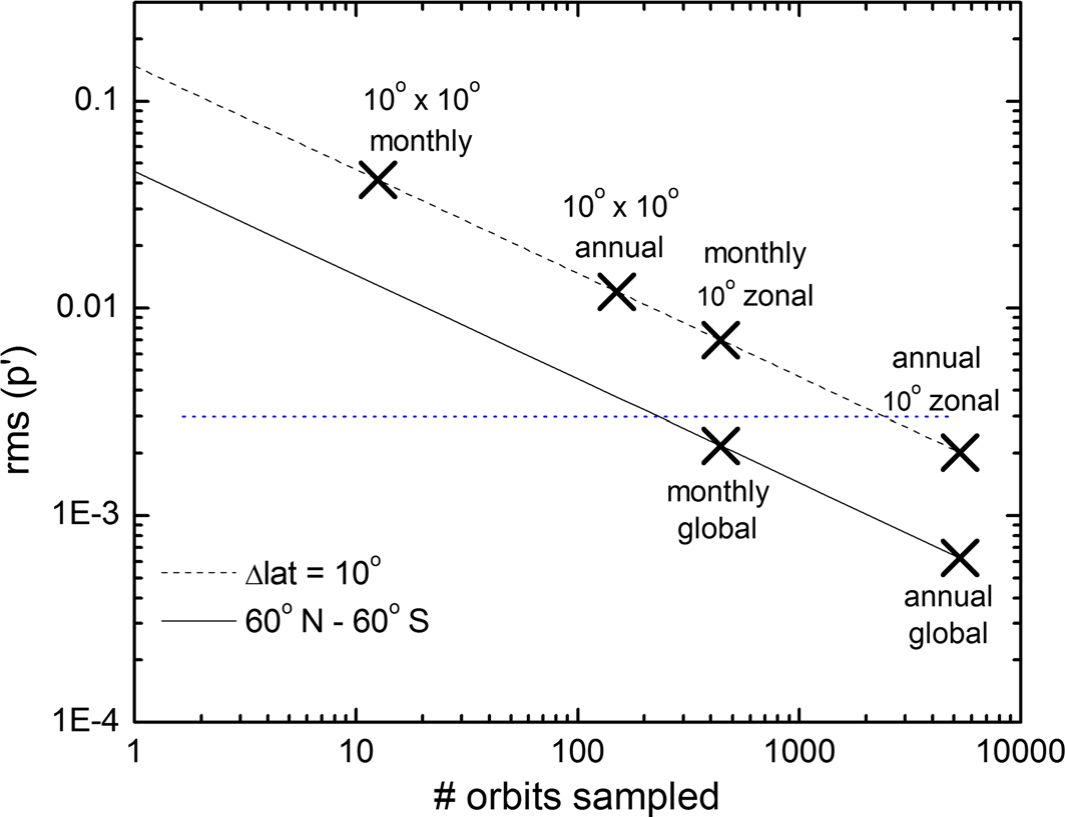
RMS uncertainty in estimated cloud fraction, *p*′, for different space–time averaging scales assuming mean cloud fraction of 50%

These characteristics of lidar result in a highly stable observing system which is referenced to fundamental benchmarks, so that overlap between successive lidar missions is not required to transfer calibrations. For nadir-viewing instruments such as lidar, sampling can be an important limitation, however. But even nadir-viewing measurements can provide accurate sampling on the space and time scales relevant to climate. Uncertainties in sampling cloud cover using a nadir-viewing sensor can be estimated using an approach from Key (1993), who develops a simple expression for the variance of an estimate of 2D cloud cover based on observations along one-dimensional transects. The method assumes a random, isotropic field of circular clouds. The variance of estimated cloud cover, *p*′, is a function of the number and length of the transects and the autocorrelation scale of cloud cover:

2 $$\text{var} \left( {p^{\prime } } \right) = 2p\left( {1 - p} \right)\frac{1 - 1/aL}{aNL}$$

where *N* is the number of transects, *L* is the length of the transects, and *a* describes the correlation scale such that *aN* is the number of independent samples. The uncertainty in estimated cloud cover decreases linearly as *aNL* increases. Assuming mean cloud cover of 50% (*p* = 0.5, which maximizes the variance) and using a cloud autocorrelation scale of 50 km, Fig. 2 shows how uncertainties in cloud cover estimates decrease as space–time sampling increases. Symbols indicate a few relevant space–time scales. This theoretical analysis predicts a RMS sampling uncertainty in global monthly mean cloud cover of 0.1%, well below the National Institute of Standards and Technology (NIST) requirement, and 1% for monthly zonal mean cloud cover with 10° latitudinal resolution.

**Fig. 3:**
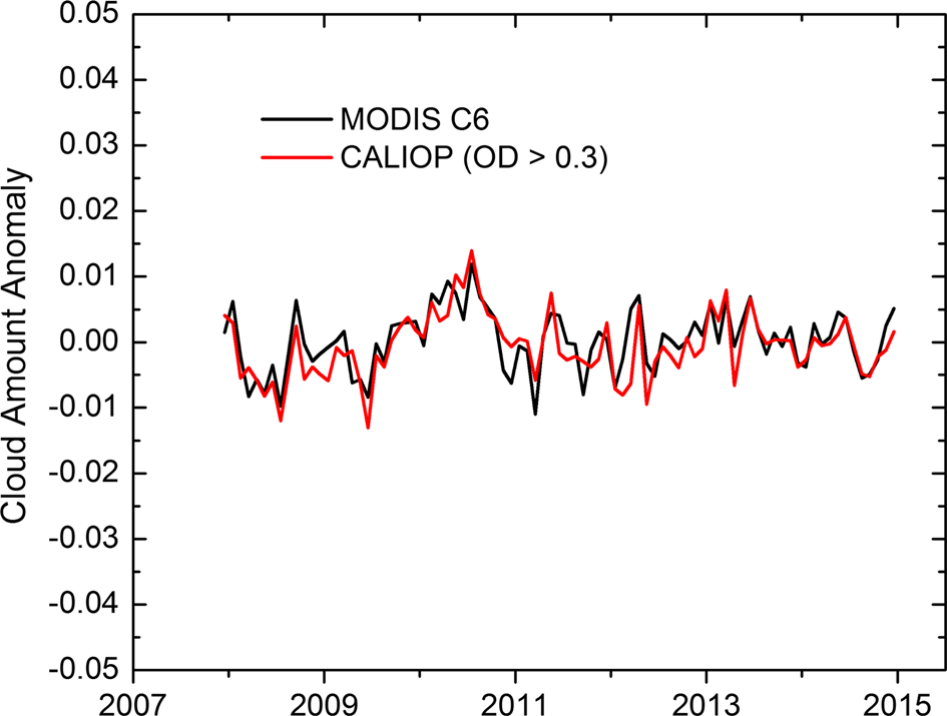
Cloud amount anomalies, 60°S–60°N, from MODIS Collection 6 full-swath observations and thresholded CALIOP nadir observations (optical depth > 0.3 only)

As a check on this theoretical estimate, Fig. 3 shows the anomalies of monthly mean cloud cover between 60°N and 60°S from nadir-viewing CALIOP observations and from full-swath Moderate Resolution Imaging Spectroradiometer (MODIS) Collection 6 data. CALIOP cloud amounts were computed using only cloud layers with optical depths greater than 0.3, in an attempt to adjust for optically thin clouds below the MODIS detection threshold. It can be seen the anomalies track closely between the two instruments with an RMS cloud fraction difference of 0.0031 and a trend which is not statistically different from zero. This result is reasonably consistent with the theoretical prediction shown in Fig. 2 (monthly global ~ 0.2%), in spite of the rather simple correction for sensitivity differences between CALIOP and MODIS. Similar behavior is seen in comparing anomalies of tropical cloud amount. These results support the ability of nadir-only observations to provide sufficient sampling to meet or exceed climate-accuracy requirements at monthly global scales and to provide sufficiently accurate estimates at monthly zonal scales.

Additionally, CALIOP has one advantage over MODIS related to sampling. Cloud detections from MODIS and many other satellite sensors rely on observations using 1 km pixels or larger. Many clouds smaller than a kilometer are found in trade cumulus regions, and it has been shown that this can lead to overestimates of cloud fraction relative to sensors having smaller footprints (Wielicki and Parker 1992; Zhao and Di Girolamo 2006). Moreover, this overestimate is sensitive to the spatial distribution of clouds so that it may be difficult to separate changes in cloud fraction from changes in the spatial organization of trade cumulus (Zhao and Di Girolamo 2006). The lidar footprint of CALIOP (about 100 m diameter) is much smaller than the 1 km pixels used for MODIS cloud detection and greatly reduces the occurrence of broken cloud within a footprint.

**Fig. 4:**
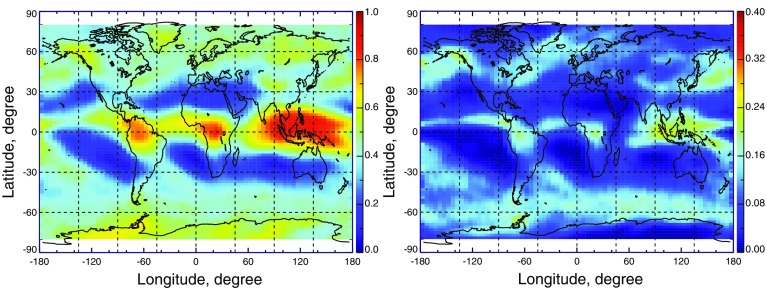
Annual mean coverage during 2008 of cirrus and ice-topped clouds (left panel) and opaque ice-topped clouds (right panel)

**Fig. 5:**
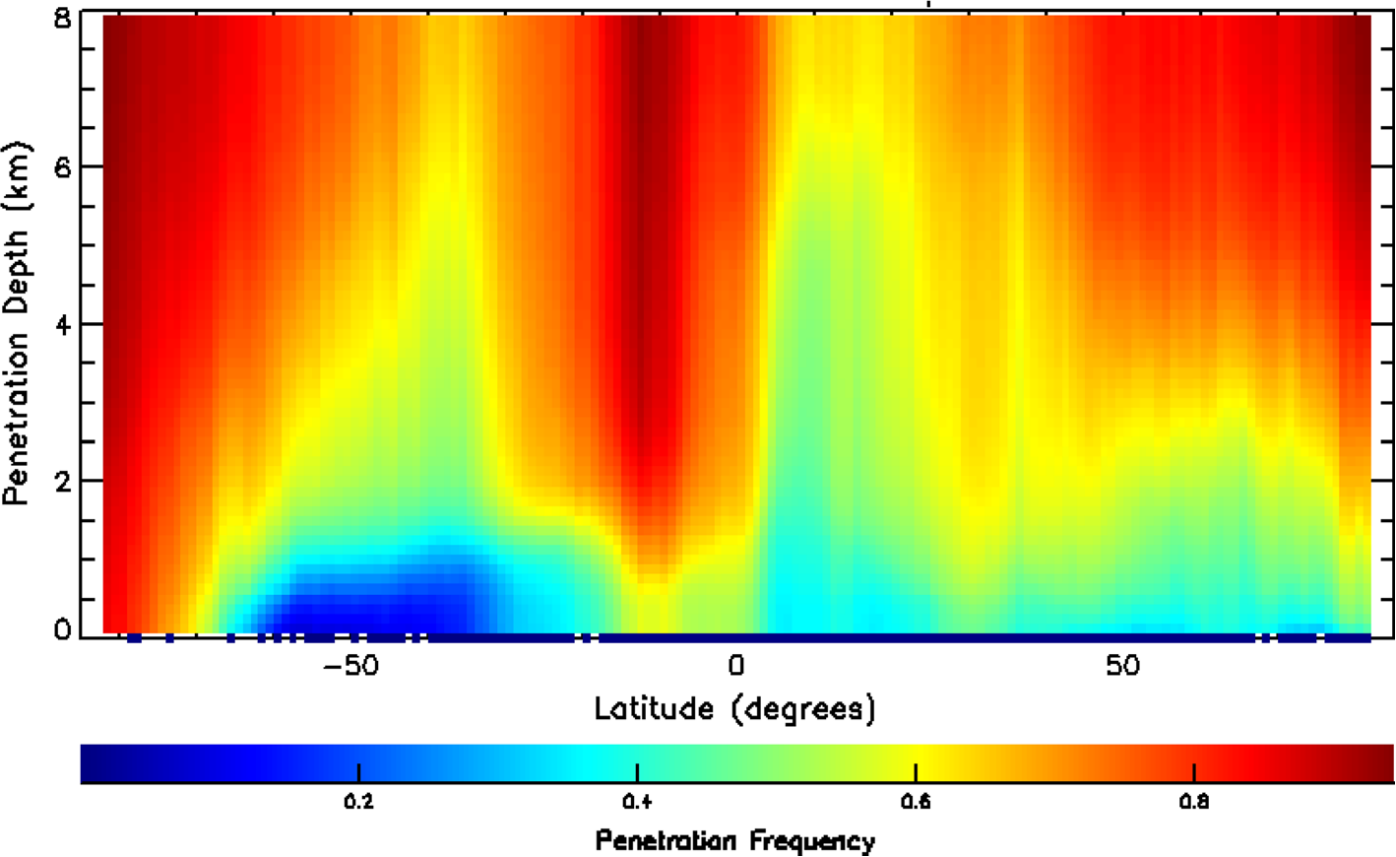
Zonal mean CALIOP frequency of penetration in cloudy columns, from TOA down to altitude *y*, for July 2009 based on single-shot 532 nm data. In the vicinity of the InterTropical Convergence Zone (ITCZ), about 20% of single shots are fully attenuated above 8 km

Another limitation of lidar is attenuation; lidar signals do not penetrate through optically thick clouds. A typical rule of thumb is that usable lidar signals penetrate to an optical depth of about 3. Because of the 100-m diameter footprint of CALIOP, relatively large for lidar, small-angle forward scattering in ice clouds provides enhanced penetration to optical depths of about 5. Figure 4 compares annual average cloud cover of all ice clouds (42%) and of ice-topped clouds which are opaque to the lidar (9.1%). The relatively rare opaque ice clouds are found mostly at mid-to-high latitudes and in regions of tropical deep convection. As a consequence, warm clouds in subsidence regions are well observed by satellite lidar. Figure 5 presents another view of lidar attenuation, showing zonal mean penetration statistics from CALIOP. The lidar signal is seen to frequently penetrate into the lower troposphere, essentially penetrating most ice cloud and being attenuated within most water clouds.

## Constraints on Cloud Feedbacks from Lidar

As discussed above, cloud feedbacks result from the small but radiatively important changes in cloud fraction, height, optical depth, or water phase in response to rising greenhouse gas concentrations. These parameters have large natural variability making the detection of forced trends difficult. The small changes in these properties which are driven by warming will only emerge from the noise of natural variability on multidecade time scales. This places stringent requirements on the long-term accuracy and stability of sensors used to detect change. Rigorous analysis shows the time required for a perfect instrument to detect a trend of magnitude |*ω*_o_| with probability of 90% is:

3 $$n = \left[ {\frac{{3.3 \sigma_{\varepsilon } }}{{\left| {\omega_{o} } \right|\left( {1 - \phi } \right) }}} \right]^{2/3}$$

where *n* is the number of years of observations required, *σ*_*ε*_^2^ is the variance of a white noise process representing the natural variability of the parameter being trended, and *ϕ* is the autocorrelation (Weatherhead et al. 1998). For a given value of *ϕ* it can be seen that the trend detection time increases as [*σ*_*ε*_/|*ω*_o_|]^2/3^. Given the long time-scales required to detect climate trends, measurement accuracy and long-term stability are more important than the precision of individual measurements, and calibration uncertainties can significantly extend the trend detection time.

Global-mean surface air temperature is expected to increase at about 0.2 K/decade over the next few decades. Wielicki et al. (2013) show that the trend uncertainty for an infrared radiometer with perfect calibration reaches 0.2 K/decade in about 12 years, but if an instrument with calibration accuracy similar to that of IASI or AIRS was used, it would take more than 20 years to reach the same trend uncertainty. Additional sources of bias such as orbit drifts or changes in channel spectral widths between instruments extend the detection time even further.

In addition to problems with instrument calibrations, passive retrievals of cloud height and optical depth are under-constrained. The forward models of the atmosphere which form the basis of cloud retrievals are highly simplified and often involve poorly justified assumptions (see discussions by Stephens and Kummerow (2007) and Pincus et al. (2012) for example). The presence of just a single cloud layer is one of the most common assumptions of passive cloud retrievals, whereas multilayer cloud occurrence ranges from 24 to 40%, depending on the definition of the gap separating single- and multilayer clouds, and as high as 60% in certain parts of the tropics (Mace et al. 2009; Matus and L’Ecuyer 2017). The most common situation is high ice cloud located over low warm cloud. Retrieval of an effective single-layer cloud height in the presence of multiple cloud layers can result in cloud height errors of as much as several kilometers (Holz et al. 2008), causing high clouds overlying low clouds to be reported as mid-level clouds. Other biases are found in the retrieval of broken cloud scenes or nighttime retrievals of low clouds where there is limited thermal contrast with the surface.

For all these reasons, evaluations of cloud vertical distributions derived from ISCCP show significant differences with CALIPSO–CloudSat (Rossow and Zhang 2010; Tselioudis et al. 2013). Comparison of joint histograms of cloud top pressure and optical depth (CP-*τ* diagrams) from ISCCP and from CALIPSO–CloudSat shows that ISCCP retrievals tend to place high clouds too low and low clouds too high (Mace and Wrenn 2013). While the ISCCP dataset provides our best long-term (30 + years) cloud observational dataset, these ambiguities and biases in the retrieved vertical distribution of clouds impact our ability to interpret the observed distribution of clouds. As an example, combining cloud profiles from CALIOP and CloudSat with other A-train observations have significantly improved previous estimates of the surface radiation budget due to improved estimates of cloud base height from the active sensors (Kato et al. 2011; Stephens et al. 2012).

To understand and model cloud feedbacks, we must be able to link changes in cloud radiative effects to the types of cloud which are changing and active sensors are the best tools we have for doing this. Evaluation of errors in CMIP5 clouds against CALIPSO–CloudSat observations has shown that errors in cloud simulation are due primarily to the cloud parameterizations used in the models rather than errors in simulating the large-scale atmospheric state (Su et al. 2013). Caldwell et al. (2013) reach a similar conclusion based on a number of recent General Circulation Model (GCM) and Large Eddy Simulation (LES) studies. Therefore, in addition to constraints on cloud radiative feedbacks, we also need observational constraints on the processes driving the changes and active sensors provide unique capabilities to do this.

Much recent work on cloud feedbacks has focused on deep convection in the tropics and clouds in regions of moderate to strong subsidence. In the following sections we focus on how active sensors can help in these specific areas of interest.

### Tropical Convective Regions

While the tropical LW cloud feedback is robustly positive in climate models, it varies substantially from weakly positive (0.2) to more than 1 W/m^2^/K (Zelinka and Hartmann 2010; Tomassini et al. 2013) and the predicted change in cloud height over the twenty-first century varies by a factor of two, from about 600 to 1100 m (Zelinka and Hartmann 2010). A decrease in tropical anvil coverage tends to accompany the cloud rise, and the changes in high cloud amount represent the primary source of diversity in the LW feedback. A “stability-iris” mechanism, which could be responsible for decreasing anvil coverage, has recently been proposed (Bony et al. 2016), but the net radiative effect of the changes in anvil coverage are ambiguous. For tropical high clouds, LW and SW CRE tend to compensate but the net balance depends on optical depth, which could change as part of changes in anvil coverage. Further, decreasing anvil coverage might induce a change in lower-level clouds via changes in downwelling radiation. Whether anvil clouds exhibit a stability-iris effect will be an important observational test of climate models and of our understanding of tropical feedback processes.

Evaluation of the predicted ascent of tropical clouds using passive observations is limited by (1) systematic errors in the long-term cloud record due to drifts in calibration and orbit drifts, and (2) ambiguities in cloud height–optical depth relationships (Mace and Wrenn 2013). Studies based on passive satellite observations have been used to confirm the underlying mechanism and that the sign of high cloud feedback is positive, but ambiguities in passive cloud datasets are too large to provide useful constraints on the magnitude of the feedback (Zelinka and Hartmann 2011; Marvel et al. 2015; Li et al. 2012; Norris et al. 2016). Shea et al. (2017) present a trend detection analysis for the global-mean difference between effective cloud top temperature and surface temperature, finding that the trend simulated by CMIP5 models could be constrained with about 12 years of observations from a perfect radiometer. Using an instrument with calibration accuracy similar to that of MODIS or Visible Infrared Imaging Radiometer Suite (VIIRS), however, requires 40–50 years of observations.

Rather than retrieving only an effective cloud height, lidar produces vertically resolved profiles of optically thin clouds and the upper portions of optically thick clouds; visible optical depth and infrared emissivity are related by:

4 $$\epsilon = 1{-}e^{ - k\tau }$$

where *τ* is the visible optical depth and *k* is the ratio of visible optical depth to 12 μm absorption optical depth. The value of *k* depends on particle size and has a weak dependence on crystal habit but for typical conditions has a value close to 1/2 (Garnier et al. 2015). As mentioned above, small-angle forward scattering in ice clouds enhances the penetration of a satellite lidar, so that CALIOP profiles the upper part of deep convective clouds to an optical depth of about 5. This corresponds to an emissivity of about 0.9 and is the part of the cloud which is relevant to radiative interactions with the top of the atmosphere—which can extend over depths of several kilometers in dissipating tropical convective systems.

All satellite sensors have limitations in characterizing clouds, and the various climatologies derived from them reflect these limitations (Stubenrauch et al. 2012). In recent years “satellite simulators” have been developed which attempt to sample model output in a way which is consistent with the limitations of the various satellite sensors which provide cloud observations (Webb et al. 2001; Bodas-Salcedo et al. 2011). These simulators provide a more consistent way of comparing observed clouds with model simulations. In particular, the “COSP/lidar simulator” (Chepfer et al. 2008, 2010) simulates the sampling limitations of the CALIPSO lidar due to nadir-only viewing and attenuation of the signal in dense clouds, as described in the previous section.

Chepfer et al. (2014) analyzed climate model simulations to show the predicted cloud rise is manifested as an upward shift in the profile of cloud occurrence. This ascending cloud profile is expected to be the first signature of the response of clouds to warming to emerge from the noise of natural variability. Use of COSP/lidar simulator output showed the signal of cloud rise can be robustly detected using satellite lidar observations, and observation of the upward shift in cloud profile is a more robust diagnostic than changes in effective cloud top heights from passive sensors (Li et al. 2012).

As a follow-on to Chepfer et al. (2014), the fraction of opaque cloud at an altitude of 6 km was defined as a parameter related to the ascent of tropical deep convection. While not an optimum metric of LW feedback, this was the best option from the available simulator output saved from CMIP5 experiments. COSP/lidar outputs from HadGEM2-A Control, and + 4 K AMIP experiments were used to determine the change in this metric over 100 years. Natural variability of the metric was derived from 7 years of CALIOP observations. While long enough to capture ENSO, the 7-year record is too short to capture longer-term modes of variability and so somewhat underestimates natural variability. Following the approach of Loeb et al. (2009), a 100-year time series of synthetic observations was constructed by adding zero-mean random fluctuations based on observed variability and autocorrelations to the linear trend estimated from model COSP/lidar output. A 200-member ensemble of 100-year time series of the metric, averaged over the tropics, was then produced, and trends were computed for each ensemble member by applying linear regression for all months between 2008 and 2108.

**Fig. 6:**
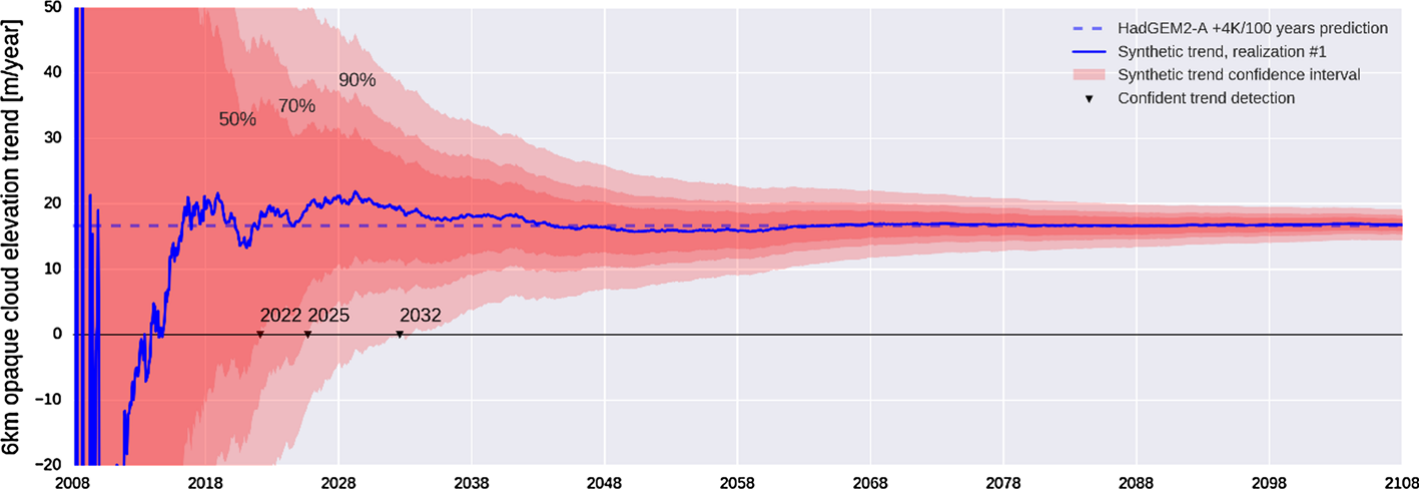
Slope of the linear regression as a function of lidar observation record length from the HadGEM-2 CALIPSO simulator output. Shading shows 50, 70, and 90% confidence envelopes

Results averaged over tropical ascent regions are shown in Fig. 6, illustrating the detectability of long-term cloud trends. Starting in 2008, the blue curve in Fig. 6 shows the mean trend in simulated lidar observations of opaque clouds computed from one ensemble member. Shading indicates the probability envelopes of trends computed from the entire ensemble. The results show the current CALIPSO record is too short to constrain even the sign of the LW feedback due to tropical ascent, but extending the record another 15 years to 2032 would provide a highly confident constraint on the sign of the feedback and a significant constraint on the magnitude. The lidar observations would of course also provide constraints on changes in coverage of high clouds.

Because of the inherent high accuracy of lidar cloud altitudes, measurements from one lidar instrument can be related to that of another without having measurement overlap. Thus, gaps between CALIPSO and future lidar missions do not represent a fundamental impediment to constructing a consistent long-term data record. The EarthCARE mission (Illingworth et al. 2015), currently scheduled for launch in 2019, will probably not last long enough to provide a significant constraint on tropical cloud ascent. Cloud heights from a future lidar system operating in the late 2020s, however, could be related to those from CALIPSO regardless of whether there are additional lidar missions in the interim period. Observations from such a future lidar mission, combined with the existing record from CALIPSO, would then provide a baseline of more than 20 years, sufficient to detect forced trends in cloud top heights as discussed in Chepfer et al. (2014) or shown in Fig. 6.

### Low-Latitude Subsidence Regions

The dominant uncertainty in the cloud feedbacks simulated by climate models is associated with shallow marine clouds—stratus, stratocumulus, and trade cumulus (Bony et al. 2006). Due to their low altitude, these clouds drive SW feedbacks more strongly than LW. Shallow marine clouds are highly sensitive to changes in their environment and so may potentially undergo significant changes as SST increases and the tropical overturning circulation weakens. Most global climate models predict a positive cloud radiative feedback from shallow marine clouds, arising from a small but radiatively significant decrease in cloud fraction, but there is significant uncertainty in the magnitude and some models predict a near-zero or somewhat negative shallow cloud feedback. Understanding the physical mechanisms behind the diversity in shallow cloud feedbacks and improving their representation in models is one of the current research priorities.

The radiative properties of these shallow clouds are the result of a complex interplay of local processes (radiative cooling, entrainment, turbulent mixing, precipitation, etc.) and interactions with the large-scale environment, taking place over a range of space and time scales (Wood 2012; Bretherton 2015). The diversity of SW cloud feedback estimates from global models is a result of the difficulty of adequately representing these processes and their interactions. Computational limitations prevent models from explicitly resolving all these processes, and they must necessarily rely on parameterizations to a greater or lesser extent. While simulations from high-resolution models tend to exhibit more consistency than global models, even large eddy simulation (LES) models still rely on parameterized cloud microphysics. More importantly, LES studies tend to be run under simplified configurations on small spatial domains, cannot realistically represent interactions with the large-scale environment, and so are limited in their ability to estimate long-term feedbacks (Vial et al. 2017).

While LES models have limitations, they have been used extensively over the last 20 years or so, along with simpler mixed-layer models, to gain insight into the mechanisms driving shallow clouds and their responses to climate change. While the dominant control on shallow clouds in the present climate is the strength of the marine inversion, LES studies indicate an increasing moisture gradient at the marine inversion with climate warming will enhance turbulent entrainment-driven drying of the marine boundary layer, leading to thinner clouds and reduced cloud cover (Bretherton 2015). Reduced radiative cooling at cloud top from increasing greenhouse gas concentrations can reinforce this cloud thinning. But this cloud thinning would be partially compensated by the expected increased temperature gradient at the marine inversion, promoting thicker clouds and increased cloud cover. Similarly, weakening of the tropical overturning circulation could induce a compensating effect via reduced subsidence, deepening of the marine boundary layer, and thicker clouds (Bretherton 2015). Overall, LES studies point to a cloud thinning in response to climate warming, representing a positive cloud feedback. A degree of consistency in these predictions has been found between simulations from different high-resolution models (Blossey et al. 2013), but LES studies are inconclusive regarding the magnitude of the shallow cloud feedback.

Given the much larger areal coverage of the trade regime relative to marine stratus, global net shallow cloud feedbacks may be driven more by the response of trade cumulus than by clouds in regions of strong subsidence. Recent research has focused on the potential role of shallow convective mixing in controlling low-cloud amount (Sherwood et al. 2014). Vial et al. (2016) found that the strength of low-level cloud feedback in a global model depended on the closure scheme adopted for the convective parameterization. More generally, the feedback strength predicted by the model was found to depend sensitively on parameterization assumptions and on subtle couplings between different boundary layer processes. Recent results from LES modeling on large domains (~ 50 km) also point to a significant role for mesoscale circulations in regulating properties of the cloud field in trade cumulus regimes (Seifert et al. 2015). These circulations occur on scales typically too small to resolve in global models and too large for standard LES domains, and are just beginning to be explored as modeling capabilities expand to better capture scale interactions.

Recent high-resolution modeling studies have shown that low-cloud feedbacks arise from a number of competing mixing and stabilization processes and that cloud vertical structure may change in response (Blossey et al. 2013; Bretherton 2015). Brient et al. (2015) find that boundary layer cloud fraction in regions of weak subsidence is primarily driven by two opposing mechanisms: shallow convective mixing which dries the lower atmosphere—reducing cloud fraction—and turbulent moistening of the boundary layer, which tends to stabilize and increase cloud fraction. They suggest that long-term changes in cloud vertical structure are associated with the processes responsible for SW shallow cloud feedbacks and show the sign of the net balance between competing stabilization and mixing processes leads to either increasing or decreasing cloud top heights. Therefore, watching how the vertical structure of shallow clouds responds to warming will provide insights into the mechanisms responsible for shallow cloud feedbacks in the real world.

**Fig. 7:**
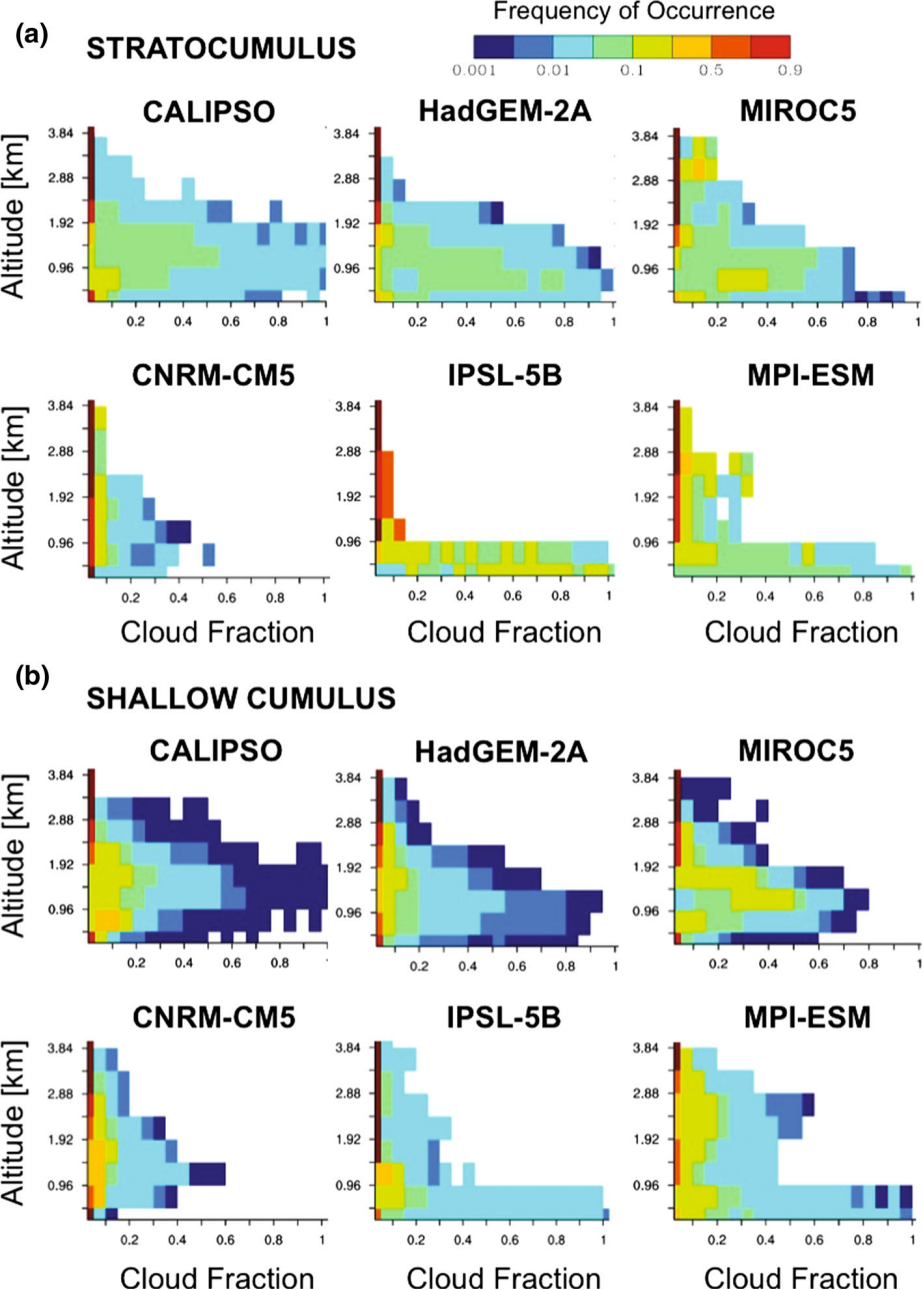
Height-resolved frequency of occurrence of cloud layers for non-overlapped low-level cloud conditions in subtropical stratocumulus and shallow cumulus regimes as observed by CALIOP and as simulated by five CMIP5 models. Adapted from Nam et al. 2012

TOA radiances provide only a weak test of how well clouds are represented in models. Lidar provides the highest accuracy and the best vertical resolution for evaluating model representations of cloud fraction and vertical development in the shallow marine boundary layer. Nuijens et al. (2015) used ground-based lidar observations from the Barbados Cloud Observatory (Stevens et al. 2016b) to evaluate trade cumulus simulated by CMIP5 models, finding the models simulated a reasonable representation of mean cloudiness in the trade-wind layer but didn’t reproduce the variability in cloudiness observed on short time-scales. In Nam et al. (2012), observations from CALIOP were used to discriminate between CMIP5 simulations of shallow marine clouds in the present climate (Fig. 7). Their evaluation is based on the altitude-resolved cloud fraction of non-overlapped low clouds from CALIOP observations, aggregated over global stratocumulus regimes (Fig. 7a) and global shallow cumulus regimes (Fig. 7b). It can be seen that the models tend to underestimate low-cloud cover and often predict stratocumulus-type clouds in regimes where shallow cumulus cloud types should prevail. Other studies have also noted that the variability of low-cloud cover in models and the relation to large-scale conditions correlate poorly with observations (Qu et al. 2015; Myers and Norris 2015). Further, in these studies the models in better agreement with observations tend to have higher ECS and/or larger cloud feedback.

**Fig. 8:**
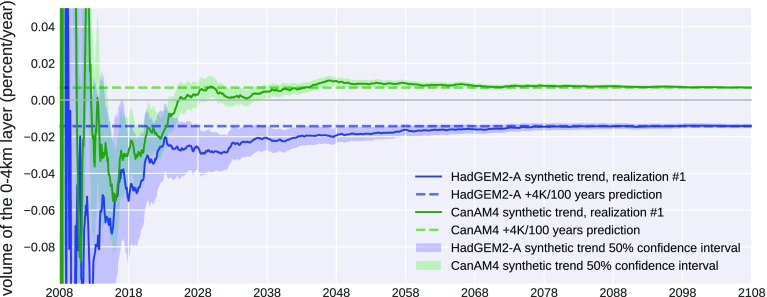
Same as Fig. 6 but for Low Cloud Volume, comparing CALIPSO simulator output from the HadGEM2 and CanAM4 models

Using the same method as described above for tropical ascent regions (Fig. 6), a trend analysis based on time series of synthetic observations was performed for tropical subsidence regions. For subsidence regions, the metric which was simulated was “Low Cloud Volume,” computed as the sum of the vertically integrated cloudy sections of lidar profiles between the ocean surface and 4 km. A change in Low Cloud Volume would be produced by either a change in boundary layer cloud coverage or a change in the boundary layer cloud vertical extent. Simulated trends of this diagnostic were constructed from two CMIP5 models in a similar way as for Fig. 6, again using output from the COSP/lidar simulator. Figure 8 shows the long-term trend of this diagnostic derived from COSP/lidar simulator outputs. The SW cloud feedbacks predicted by these two models are significantly different from each other and trends of the low-cloud diagnostic start to separate in the early 2020s. By the late 2020s the trends separate with some confidence and would provide a test of model performance.

The above discussion points to the need to better understand shallow cloud processes and improve model parameterizations. Satellite retrievals of cloud properties (cloud height, albedo, phase, etc.) can be used to gain insight into the processes responsible for cloud responses to environmental changes. Satellite lidar is a relatively new tool to observe cloud properties: In addition to cloud top height and altitude-resolved cloud fraction, lidar can detect the height of the trade inversion in clear-sky conditions when there is a gradient in aerosol concentration across the inversion. Direct observation of the small predicted changes in height of the marine inversion (Blossey et al. 2013) could provide insight into the processes underlying observed changes in shallow clouds. In addition to lidar, passive radiometers and cloud profiling radar are needed to provide complementary measurements of liquid water path, droplet size, and precipitation—all necessary to identify and characterize the processes responsible for the changes in cloud radiation.

One critical parameter which is not currently retrieved is the geometric thickness of shallow clouds—particularly stratiform boundary layer clouds—which would provide a valuable constraint on cloud processes (Wood 2007). A cloud thickness retrieval would also provide an observational test of cloud adiabaticity. For an adiabatic cloud, a relation between cloud optical depth, cloud droplet number concentration (CDNC) and cloud thickness can be derived from theory. Cloud optical depth and CDNC are currently retrieved from imagers such as MODIS. Addition of an independently retrieved cloud thickness would give an observational test of the adiabaticity relationship. Cloud thickness combined with lidar cloud top heights would give cloud base height and an estimate of the lifting condensation level, also providing insight into the thermodynamic state of the marine boundary layer (Stevens 2007). Retrieval of cloud thickness from airborne multiple-field-of-view lidar (Cahalan et al. 2005) has been demonstrated and the feasibility of implementing this capability in a future satellite lidar should be pursued.

### Middle and High Latitudes

This paper has focused on cloud feedbacks from low-latitude clouds, but there has also been interest in shortwave optical depth feedbacks at mid- and high latitudes. Climate models are consistent in predicting that extratropical clouds will become optically thicker and brighter due to a combination of increases in total water path and changes of thermodynamic phase from ice to water. A number of recent studies have looked for support for these predictions in the satellite record, primarily relying on passive observations from the ISCCP, MODIS and CERES datasets (Ceppi et al. 2016; Gordon and Klein 2014; Terai et al. 2016). While observational uncertainties and disagreements between datasets have prevented quantitative estimates of the cloud optical depth feedback, observations and models are found to be in qualitative agreement that the sign of this feedback is negative.

Changes of thermodynamic phase from ice to liquid are potentially an important driver of these optical depth feedbacks. Lidar is able to directly observe cloud thermodynamic phase (via range-resolved backscatter depolarization signatures) in the first few optical depths below cloud top (Hu et al. 2009). These observations are far more accurate and useful than from passive techniques (Jin and Nasiri 2014; Cho et al. 2009) and so provide a much stronger test of models than passive observations. Comparisons with CALIOP observations of cloud phase have shown that models generally tend to simulate too little supercooled liquid water and too readily convert liquid to ice (Cesana et al. 2015).

The Southern Ocean mid-latitude storm tracks have been a region of particular interest, where climate models tend to exhibit significant biases in downwelling SW surface radiation. These SW radiation biases have been linked to the representation of shallow marine clouds in the cold sector of extratropical storms. CALIOP observations have shown a lack of supercooled water in these regions in models, due to biases in the partitioning of cloud water between liquid and ice (Bodas-Salcedo et al. 2014, 2016; Forbes et al. 2016). These biases in how models partition liquid and ice also have implications for model estimates of climate sensitivity (Tan et al. 2016). Continuation of lidar cloud phase observations is essential for monitoring trends in cloud phase driven by climate warming.

### Aerosols as a Cloud Controlling Factor

Cloud cover or albedo might also change in response to changing aerosol concentrations. While aerosols are not thought to play a significant role in cloud feedbacks, simple conceptual models (Twomey 1977; Albrecht 1989) make clear the potential of aerosol microphysical and radiative effects to influence cloud properties. In the real world, however, numerous other microphysical and macrophysical processes likely act to reduce the response of cloud to changing aerosols predicted by these simple conceptual models (Stevens and Feingold 2009; Seifert et al. 2015). Influences of aerosol on cloud also depend on the vertical location of the aerosol relative to clouds (Amiri-Farahani et al. 2017), which can change even the sign of the effect. Given these uncertainties, the magnitude of long-term trends in cloud properties due to aerosol influences is highly uncertain.

If the changes in aerosol concentrations are due to changes in anthropogenic emissions this would represent an aerosol indirect forcing, whose effects might be mistakenly attributed to cloud feedbacks (Gettelman et al. 2016). If emissions of natural aerosols change in response to warming surface temperatures and then alter cloud properties, this could be thought of as a cloud-mediated aerosol feedback (Carslaw et al. 2010). To control for these potential aerosol-induced effects on clouds requires, at a minimum, vertically resolved monitoring of trends in aerosol loading. With regard to the long-term monitoring of aerosols, passive aerosol retrievals are very sensitive to calibration drifts and can be biased by a variety of cloud-induced artifacts including cloud masking errors and side-scattering of sunlight from clouds into cloud-free columns (Várnai and Marshak 2009). If cloud fraction changes over time, these cloud-induced aerosol retrieval artifacts could produce spurious trends in aerosol, leading to mistaken attribution of some of the observed cloud changes to aerosol indirect effects. Lidar has more stable long-term calibration, is less susceptible to cloud-induced artifacts and would provide a higher confidence indication of long-term aerosol change, in addition to providing a vertically resolved measurement rather than just a column integral. Satellite lidar is also able to retrieve cloud top extinction and cloud droplet number concentration (Zeng et al. 2014). Retrieval of droplet size at cloud top is likely also possible, though still under development at this point. These retrievals can be performed with even a simple elastic backscatter lidar (such as CALIOP) but satellite High Spectral Resolution Lidar (HSRL, She et al. 1992; Hair et al. 2008) would provide the additional advantage of direct measurement of aerosol extinction.

## Meeting the Observational Challenge

The availability of colocated observations from cloud lidar and cloud profiling radar in the A-train have spurred substantial advances in our understanding of cloud-climate processes. The existing 11-year record from CALIOP and the A-train represents an initial baseline sufficient to characterize the current climate state (Chung et al. 2012) but is not yet long enough to fully capture interannual variability or to detect climate trends. Continued advancement of our understanding of cloud processes and cloud feedbacks depends on our ability to continue the types of measurements that CALIPSO, CloudSat and the A-train have provided. Observing the small forced trends in cloud height predicted by models and proper attribution of changes in CRE to cloud type requires cloud profiling from radar and lidar. Sustained observation by a small suite of core instruments is necessary to advance beyond the current state of understanding achieved from the A-train and globally monitor the evolution of clouds as they begin to move outside the envelope of current variability.

In addition to monitoring changes in clouds, we need to understand and derive observational constraints on the processes responsible for those changes. Ongoing observations are needed to understand and constrain physical mechanisms responsible for cloud-climate-feedbacks which are now being proposed (e.g., Hartmann and Larson 2002; Sherwood et al. 2014; Bony et al. 2016). The ability of lidar to accurately observe changes in the vertical distribution and coverage of clouds is part of the essential capabilities required.

Table 2 outlines a set of continued satellite observations to address the issues which have been discussed above. A minimum set would be lidar plus cloud profiling radar and a passive radiometer covering key channels in the visible and thermal infrared. This assumes that crucial monitoring of fundamental integral constraints on the overall energy budget—such as TOA broadband radiative fluxes and sea surface altimetry for ocean heat content– are available from sensors flying on other platforms. These measurements of integral constraints would not need to be colocated with the cloud sensors. Together, these would constitute the long-term observing system necessary to understand, quantify, and constrain cloud feedbacks.

**Table 2:** Summary of observation requirements for cloud feedbacks and colocation needs

Science objective	Geophysical variable	Sensors	Spatially matched?
LW cloud feedbacks			
	TOA LW radiative flux	Broadband flux radiometer	No
	Cloud profiles	Lidar and W-band radar	Yes
	Cloud emissivity	Thermal IR radiometer, lidar	Yes
	Water vapor and temperature profiles	AIRS/AMSR, GPS-Radio Occultation	Yes
SW cloud feedbacks			
	TOA SW radiative flux	Broadband flux radiometer	No
	Light precipitation	W-band radar	Yes
	Cloud albedo, OD, Re	Visible radiometer	Yes
	Cloud top properties	Visible radiometer, lidar	Yes
	Liquid water path	Visible and microwave radiometers	Yes
	Cloud top height, thickness, phase	Lidar	Yes
Aerosol–cloud interactions (in addition to measurements above)			
	Aerosol near cloud base	Lidar (backscatter or HSRL)	Yes

With the A-train era soon drawing to a close, follow-on lidar missions are necessary to extend the initial data record established by CALIOP. Global active profiling will be continued by the ESA EarthCARE mission (Illingworth et al. 2015), currently scheduled to launch in 2019. There will likely be a gap between CALIPSO and EarthCARE and, given the length of time required to develop new satellite missions, it may be already too late to avoid a gap between EarthCARE and a follow-on mission. However, if future lidar instruments are designed with repeatability in mind—with instrument characteristics and orbits designed to provide consistency with the existing CALIOP record—then mission overlap is not strictly required due to the inherent accuracy and stability of the primary cloud measurements, as discussed above. However, as of this writing, there are no plans within ESA or the national space agencies to continue—much less improve—this vital observational record after EarthCARE.

The focus of this paper has been on the use of active sensors to characterize the response of clouds to a warming climate. A decade of observation by satellite-borne active sensors has transformed our understanding of the vertical distribution of condensate, especially cloud ice, but these active measurements are equally important to understanding the coupling of clouds to circulation. The sensitivity of cloud feedbacks to patterns of SST change, discussed above, demonstrates the important influence of large-scale dynamics on the cloud response to warming. Experience with the A-train has demonstrated that cloud profiling from active sensors is essential to describing the vertical distribution of radiative and latent heating (Haynes et al. 2013), which are the primary ways that clouds interact with the large-scale circulation. Sustained observations of atmospheric heating derived from cloud profiling would help our understanding of processes responsible for the large-scale structure of atmospheric circulations systems, such as the jets, storm tracks and rain bands. Continuing active measurements beyond the A-train and EarthCARE is essential to our ability to understand regional climate change and to develop adaptive strategies (Bony et al. 2015).
